# Expression of Concern: The role of *CRP* and *ATG9B* expression in clear cell renal cell carcinoma

**DOI:** 10.1042/BSR-2017-1082_EOC

**Published:** 2023-04-06

**Keywords:** ATG9B, cell apoptosis, clear cell renal cell carcinoma CCRCC, CRP

The Editorial Office has been made aware of potential issues surrounding the scientific validity of this paper, hence has issued an expression of concern to notify readers whilst the Editorial Office investigates. It has been noted that the Figure 3A control panel seems to also appear as the Figure 4A UA panel from Li et al. 2017 (doi: 10.18632/oncotarget.22133). The Figure 4B GAPDH western blot bands also seem to appear as the Figure 12D A549/PTX/B-actin bands from Su et al. 2018 (doi: 10.1002/cam4.1294) and as the Figure 2E SCC-9/GAPDH bands from Wang et al. 2019 (doi: 10.1016/j.biopha.2017.04.050).

